# Association between life’s crucial 9 and kidney stones: a population-based study

**DOI:** 10.3389/fmed.2025.1558628

**Published:** 2025-03-06

**Authors:** Xiao-ran Li, Han-lin Liu, Li Wang, Jian-wei Yang, Kang-yu Wang, Si-yu Chen, Li Yang

**Affiliations:** ^1^The Second Hospital and Clinical Medical School, Lanzhou University, Lanzhou, China; ^2^Cuiying Biomedical Research Center, Lanzhou University Second Hospital, Lanzhou, China

**Keywords:** kidney stone, Life’s crucial 9, NHANSE, cardiovascular health, depression

## Abstract

**Purpose:**

This study examined the association between the Life’s Crucial 9 (LC9) score and kidney stone prevalence in U.S. adults.

**Methods:**

Using data from the National Health and Nutrition Examination Survey (NHANES) spanning 2007 to 2014, this cross-sectional analysis focused on adult participants with kidney stone onset or recurrence. LC9 scores were divided into four quartiles for analysis. Weighted multivariable logistic regression, restricted cubic spline (RCS) modeling, threshold effect analysis, and subgroup analyses were employed to evaluate the relationship between LC9 scores and kidney stone prevalence.

**Results:**

The study included 24,669 participants with an average age of 46.05 ± 0.34 years and a mean LC9 score of 73.76 ± 0.25. The overall prevalence of kidney stones was 8.45%, while the average recurrence rate stood at 2.96%. Importantly, for each one-point increase in the LC9 score, the incidence of kidney stones dropped by 1.2% (95% CI: 0.979 to 0.997, *p* = 0.014). Compared to the lowest quartile (Q1), the Q4 group exhibited a 0.305-fold higher recurrence rate (95% CI: 0.159 to 0.586, *p* < 0.001). Interaction analysis showed that race and gout significantly influenced the relationship between the LC9 score and kidney stone risk. Additionally, curve fitting and threshold effect analysis demonstrated a nonlinear association between LC9 scores and kidney stone recurrence, with a breakpoint identified at 72.777.

**Conclusion:**

An elevated LC9 score correlates with a lower risk of both kidney stone formation and recurrence. Maintaining an optimal LC9 score could be an effective approach for preventing kidney stones.

## 1 Introduction

Kidney stones are a prevalent urological condition, impacting around 10–12% of the global population, with incidence rates steadily increasing in recent decades (1). In the United States, the prevalence has grown from 3.8% in the 1970s to nearly 10% in recent years, disproportionately affecting men and older adults (2). Metabolic factors, including obesity, diabetes, hypertension, and a higher occurrence of cardiovascular events, are closely linked to kidney stone formation (3). Previous research has shown a bidirectional link between kidney stone formation and cardiovascular health (CVH) (4). Life’s Essential 8 (LE8), developed by the American Heart Association, includes eight critical health factors: diet, physical activity, nicotine exposure, sleep health, body mass index, blood lipids, glucose levels, and blood pressure. These factors are widely used to assess CVH (5). Recently, Life’s Crucial 9 (LC9) was introduced as an expansion of the LE8 framework by adding mental health as a crucial component (6). This adjustment recognizes the significant role that mental health, particularly depression, plays in influencing cardiovascular health and overall well-being. The LC9 score, therefore, includes the same eight factors as LE8, along with the addition of depression, making it a more comprehensive measure of overall health.

The link between LC9 and both the incidence and recurrence of kidney stones has yet to be fully explored. To address this, we analyzed data from the extensive National Health and Nutrition Examination Survey (NHANES) to explore how the LC9 score affects the prevalence and recurrence of kidney stones, thereby building on the relationship between cardiovascular health and kidney stones.

## 2 Methods

NHANES evaluates the health and nutritional status of U.S. children and adults through a stratified, multistage sampling method (7). Conducted by the Centers for Disease Control (CDC), the survey collects vital data on demographics, diet, and health. The studies involving human participants were reviewed and approved by the National Center for Health Statistics (NCHS) Ethics Review Board. Informed consent was obtained from all individual participants enrolled in the study. This study follows Strengthening the Reporting of Observational Studies in Epidemiology (STROBE) Statement.

### 2.1 Study population

**Figure 1:**
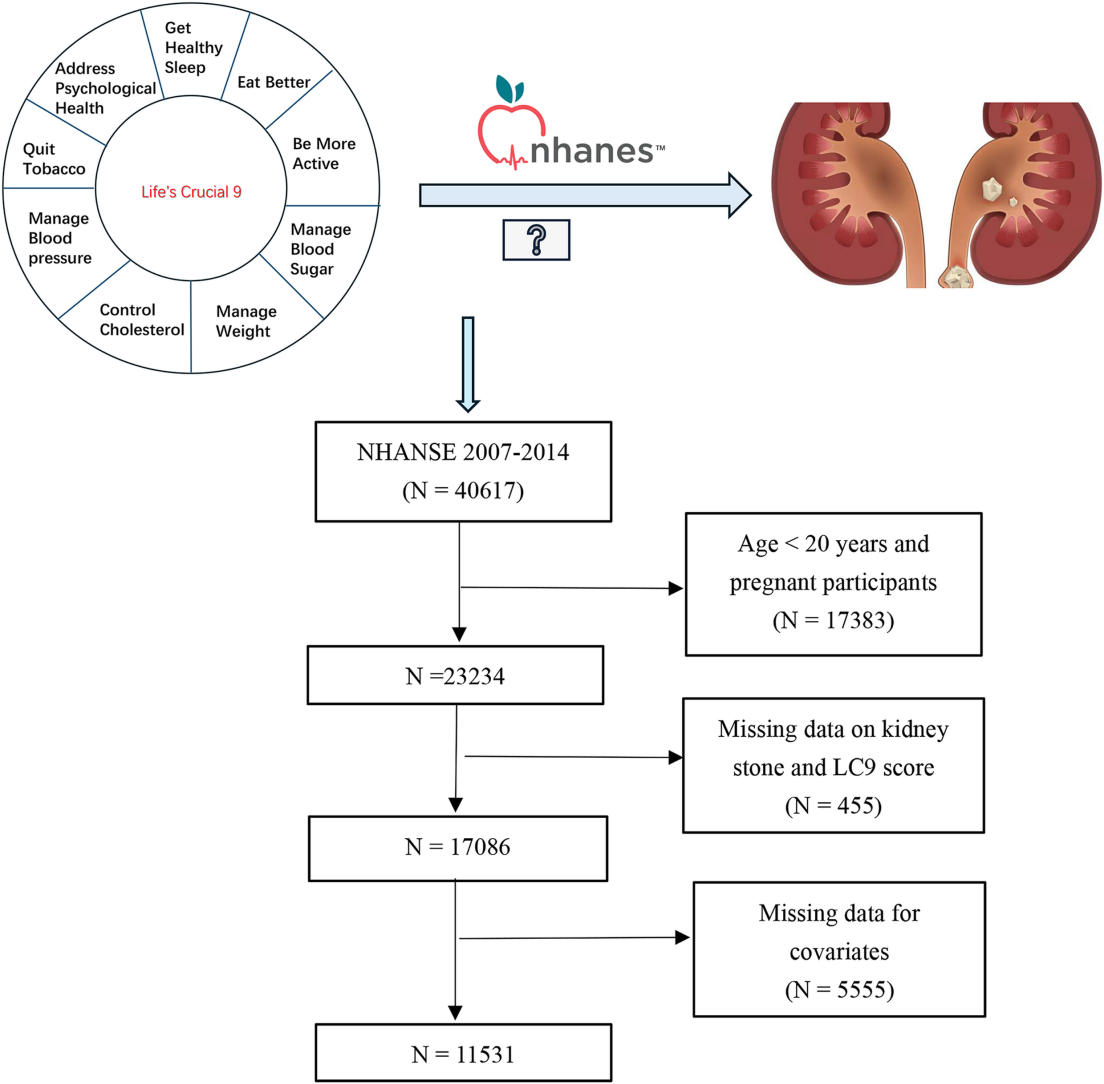
Flowchart of the study population.

We initially analyzed NHANES data from 2007 to 2014, which included 40,617 participants. The study excluded several groups: individuals under 20 years old and pregnant participants (*n* = 17,383), those missing data on kidney stones or LC9 scores (*n* = 455), and participants with incomplete covariate information (*n* = 5,555). Ultimately, the final analysis comprised 11,531 participants (Figure 1).

### 2.2 Assessment of LC9 score and nephrolithiasis

The LC9 score was calculated as the average of the eight LE8 components and the depression score (8). The depression score, derived from the Patient Health Questionnaire-9 (PHQ-9), classifies depressive symptoms into scores of 100, 75, 50, 25, and 0, corresponding to PHQ-9 ranges of 0–4, 5–9, 10–14, 15–19, and 20–27, respectively (9). The LE8 score includes four health behaviors—diet, physical activity, nicotine exposure, and sleep duration—and four health factors: BMI, lipids, blood glucose, and blood pressure. Dietary data were collected through a 24-h diet recall and assessed using the Healthy Eating Index 2015 (HEI-2015) (10). Data on physical activity, nicotine exposure, sleep duration, and diabetes status were obtained from questionnaires, while laboratory and MEC data were used to measure lipids, blood glucose, weight, height, and blood pressure. Supplementary Table S1 provides detailed information on the LE8 scoring algorithms used in the NHANES data.

The diagnosis of nephrolithiasis was determined by asking participants, “Have you/the sample person ever had nephrolithiasis?” Those who answered “yes” were classified as having a history of the condition. Participants who reported experiencing nephrolithiasis two or more times in response to the question, “How many instances of nephrolithiasis did you experience?” were categorized as having recurrent nephrolithiasis. This diagnostic method is consistent with prior research (11).

### 2.3 Covariates

We gathered data through standardized questionnaires and measurements, adjusting for multiple covariates to enhance the robustness of our analysis. Demographic variables included age, gender, race, education level, marital status, and the poverty-to-income ratio (PIR). Lifestyle factors encompassed smoking and alcohol consumption, categorized as: Never (fewer than 12 lifetime drinks), Ever (12 or more drinks in a single year or lifetime but not in the past year), and Now (currently consuming 12 or more drinks annually). Physical activity was measured by task intensity in metabolic equivalent (MET) minutes and classified as either <500 or ≥ 500 MET minutes per week. Health-related variables included BMI, hypertension, diabetes, cardiovascular diseases (CVD), and self-reported histories of gout and cancer (12).

### 2.4 Statistical analyses

Following NHANES guidelines, we combined four survey cycles using complex sampling weights (MEC examination weight). Statistical analyses included survey-weighted logistic regression for continuous variables (mean and standard error, SE) and survey-weighted chi-square tests for categorical variables (counts and percentages). To investigate the relationship between LC9 score—analyzed as both a continuous and categorical variable (quartiles)—and kidney stone occurrence and recurrence rates, we applied multinomial logistic regression across three models: Model 1 (unadjusted), Model 2 (adjusted for age, sex, and race), and Model 3 (further adjusted for marital status, PIR, education, smoking, alcohol use, diabetes, BMI, hypertension, CVD, energy intake, physical activity, gout, and cancer). Trend tests were also conducted. We used restricted cubic spline (RCS) regression to explore the relationship between LC9 scores and kidney stone prevalence and recurrence. RCS is a technique that allows us to model complex, non-linear relationships by using multiple curve segments. This method helps us understand how changes in LC9 scores might have varying effects at different levels of the score, rather than assuming a simple straight-line relationship, with nodes placed at the 10th, 50th, and 90th percentiles of the LC9 score distribution. Additionally, we applied piecewise linear regression to investigate whether the effect of LC9 on kidney stone recurrence changes at a certain threshold. This means we tested whether there is a specific point, called the ‘inflection point,’ where the relationship between LC9 scores and kidney stone recurrence shifts significantly. This approach helps to identify potential breaks in the trend that would not be captured by a simple linear model. Interaction tests assessed heterogeneity between subgroups. All statistical analyses were performed using R version 4.3, with a *p*-value <0.05 considered statistically significant.

## 3 Results

### 3.1 Participants’ baseline characteristics

Table 1 displays the weighted demographic characteristics of 24,669 participants stratified by LC9 quartiles, with 51.22% male and 48.78% female. The average age was 46.05 ± 0.34 years. Compared to Q1, individuals in the Q4 group were younger, had lower BMI, fewer instances of divorce, separation, or widowhood, higher education levels, and greater financial stability. Although smoking rates were relatively low, alcohol consumption was higher, and the prevalence of underlying diseases was lower. Higher LC9 scores were linked to a reduced risk of kidney stone occurrence and recurrence.

**Table 1:** Characteristics of participants by LC9 score quartiles: NHANES 2007–2014.

Characteristic	All *N* = 24,669	Q1 (18.88, 63.88 pts) *N* = 2,917	Q2 (63.88, 72.77 pts) *N* = 2,900	Q3 (72.77, 81.11 pts) *N* = 2,664	Q4 (81.11, 100 pts) *N* = 3,050	*P* value
**Age (years)**	46.05 (0.34)	48.87 (0.38)	48.54 (0.48)	45.86 (0.50)	42.26 (0.57)	< 0.0001
**Age category (%)**						< 0.0001
20–39	4,240 (38.11)	775 (28.35)	890 (31.70)	992 (38.78)	1,583 (49.50)	
40–59	3,981 (39.44)	1,192(46.55)	1,039 (41.55)	875 (38.49)	875 (33.57)	
≥60	3,310 (22.45)	950 (25.10)	971 (26.75)	797 (22.73)	592 (16.93)	
**PIR**	3.12 (0.05)	2.56 (0.07)	3.03 (0.05)	3.20 (0.06)	3.52 (0.07)	< 0.0001
**PIR category (%)**						< 0.0001
≤1.3	3,414 (19.66)	1,174 (30.14)	892 (20.26)	704 (17.56)	644 (13.55)	
1.3–3.5	4,141 (34.07)	1,061 (37.33)	1,095 (37.32)	967 (34.34)	1,018 (28.98)	
>3.5	3,976 (46.27)	682 (32.53)	913 (42.42)	993 (48.09)	1,388 (57.47)	
**BMI (kg/m** ^**2**^ **)**	28.44 (0.10)	32.79 (0.20)	30.12 (0.14)	27.58 (0.14)	24.76 (0.09)	< 0.0001
**BMI category (%)**						< 0.0001
<25 kg/m^2^	3,510 (31.79)	308 (10.63)	581 (18.11)	860 (31.12)	1761 (58.00)	
25–30 kg/m^2^	3,946 (35.05)	752 (25.69)	1,026 (34.93)	1,117 (44.24)	1,051 (34.34)	
≥30 kg/m^2^	4,075 (33.16)	1857 (63.67)	1,293 (46.96)	687 (24.65)	238 (7.65)	
**Physical activity (MET-minutes/week)**	4448.55 (96.57)	4445.60 (154.40)	4824.77 (169.38)	4565.41 (170.85)	4055.94 (132.73)	0.001
**Physical activity (%)**						< 0.0001
<500 MET-minutes/week	1977 (16.01)	803 (27.89)	554 (18.69)	354 (13.63)	266 (7.51)	
≥500 MET-minutes/week	9,554 (83.99)	2,114 (72.11)	2,346 (81.31)	2,310 (86.37)	2,784 (92.49)	
**LC9 score**	73.76 (0.25)	56.09 (0.17)	68.92 (0.07)	76.87 (0.06)	87.47 (0.12)	< 0.0001
**Gender**						< 0.0001
Female	5,521 (48.78)	1,349 (47.54)	1,274 (43.11)	1,203 (46.86)	1,695 (55.71)	
Male	6,010 (51.22)	1,568 (52.46)	1,626 (56.89)	1,461 (53.14)	1,355 (44.29)	
**Race (%)**						< 0.0001
Mexican American	1,516 (7.22)	356 (6.96)	391 (7.38)	387 (7.99)	382 (6.67)	
Non-Hispanic black	2,251 (9.70)	806 (15.93)	617 (10.80)	467 (8.54)	361 (5.40)	
Non-Hispanic white	5,654 (72.47)	1,332 (67.72)	1,434 (72.97)	1,323 (72.94)	1,565 (75.00)	
Other Hispanic	1,047 (4.67)	261 (4.98)	252 (4.18)	250 (4.71)	284 (4.82)	
Other race	1,063 (5.95)	162 (4.41)	206 (4.68)	237 (5.84)	458 (8.11)	
**Marital (%)**						< 0.0001
Divorced/Separated/Widowed	2,265 (16.59)	803 (25.61)	632 (19.77)	483 (14.56)	347 (9.37)	
Married/Living with a partner	6,979 (64.31)	1,641 (58.61)	1756 (63.99)	1,680 (67.04)	1902 (66.37)	
Never married	2,287 (19.10)	473 (15.78)	512 (16.24)	501 (18.40)	801 (24.26)	
**Education (%)**						< 0.0001
Less than 9th grade	786 (3.44)	257 (4.93)	211 (3.65)	185 (3.31)	133 (2.33)	
9-11th grade	1,497 (9.78)	580 (16.84)	397 (11.28)	315 (8.39)	205 (4.78)	
High school graduate	2,544 (21.15)	793 (28.47)	781 (26.72)	555 (20.99)	415 (11.72)	
Some college	3,568 (32.01)	908 (33.94)	914 (34.20)	856 (33.03)	890 (28.08)	
College or above	3,136 (33.62)	379 (15.81)	597 (24.15)	753 (34.28)	1,407 (53.09)	
**Smoke (%)**						< 0.0001
Former	2,807 (24.76)	749 (25.16)	819 (28.44)	711 (27.86)	528 (19.06)	
Never	6,385 (56.09)	946 (31.21)	1,401 (48.01)	1,574 (57.35)	2,464 (78.88)	
Now	2,339 (19.15)	1,222 (43.63)	680 (23.55)	379 (14.79)	58 (2.06)	
**Alcohol (%)**						< 0.0001
Former	1864 (13.42)	668 (20.03)	543 (15.91)	380 (11.93)	273 (7.99)	
Never	1,380 (9.56)	293 (8.23)	335 (9.36)	310 (8.82)	442 (11.23)	
Now	8,287 (77.02)	1956 (71.73)	2022 (74.73)	1974 (79.25)	2,335 (80.77)	
**Diabetes (%)**						< 0.0001
No	8,891 (81.59)	1734 (64.86)	2,145 (77.38)	2,197 (85.59)	2,815 (93.41)	
Yes	2,640 (18.41)	1,183 (35.14)	755 (22.62)	467 (14.41)	235 (6.59)	
**Hypertension (%)**						< 0.0001
No	7,088 (65.82)	1,153 (42.34)	1,556 (55.21)	1798 (71.30)	2,581 (86.29)	
Yes	4,443 (34.18)	1764 (57.66)	1,344 (44.79)	866 (28.70)	469 (13.71)	
**CVD (%)**						< 0.0001
No	10,565 (93.37)	2,516 (88.32)	2,629 (92.53)	2,479 (94.51)	2,941 (96.65)	
Yes	966 (6.63)	401 (11.68)	271 (7.47)	185 (5.49)	109 (3.35)	
**Gout (%)**						< 0.0001
No	11,067 (96.39)	2,732 (93.69)	2,757 (95.44)	2,569 (97.19)	3,009 (98.40)	
Yes	464 (3.61)	185 (6.31)	143 (4.56)	95 (2.81)	41 (1.60)	
**Cancer (%)**						< 0.0001
No	10,472 (90.27)	2,613 (88.08)	2,608 (89.67)	2,422 (90.66)	2,829 (91.95)	
Yes	1,059 (9.73)	304 (11.92)	292 (10.33)	242 (9.34)	221 (8.05)	
**Nephrolithiasis (%)**						0.01
No	10,547 (91.55)	2,594 (88.88)	2,610 (89.69)	2,443 (91.99)	2,900 (94.55)	
Yes	984 (8.45)	323 (11.12)	290 (10.31)	221 (8.01)	150 (5.45)	
**Nephrolithiasis recurrence (%)**						< 0.0001
No	11,208 (97.04)	2,801 (95.45)	2,799 (96.11)	2,592 (97.13)	3,016 (98.82)	
Yes	323 (2.96)	116 (4.55)	101 (3.89)	72 (2.87)	34 (1.18)	

PIR, poverty income ratio; BMI, body mass index; CVD, cardiovascular disease.

### 3.2 Multivariate regression analysis

In the fully adjusted model, each one-point increase in LC9 was linked to a 1.2% reduction in kidney stone risk (95% CI: 0.979–0.997, *p* = 0.014). Quartile analysis revealed that participants in the highest LC9 quartile (Q4) had a 0.636-fold lower risk of kidney stones compared to those in the lowest quartile (Q1). For kidney stone recurrence, model 3 indicated non-significant results when LC9 was treated as a continuous variable, suggesting a potential nonlinear association. However, quartile analysis showed that individuals in Q4 had a 0.305-fold lower recurrence rate than those in Q1 in the fully adjusted model (*p* for trend = 0.006) (Table 2).

**Table 2:** Association of LC9 score with nephrolithiasis.

Exposure	OR (95%CI), *p* value		
	Model 1^a^	Model 2^b^	Model 3^c^
**Nephrolithiasis**			
LC9 (continuous)	0.978 (0.973,0.984) **<0.0001**	0.980 (0.974,0.985) **<0.0001**	0.988 (0.979,0.997) **0.014**
LC9 (in quartiles)			
Quartile 1	Ref	Ref	Ref
Quartile 2	0.919 (0.718,1.175) 0.493	0.876 (0.685,1.120) 0.284	0.954 (0.750,1.214) 0.695
Quartile 3	0.696 (0.566,0.858) **<0.001**	0.692 (0.565,0.848) **<0.001**	0.816 (0.613,1.087) 0.158
Quartile 4	0.461 (0.365,0.581) **<0.0001**	0.489 (0.386,0.621) **<0.0001**	0.636 (0.461,0.877) **0.007**
*P* for trend	**<0.0001**	**<0.0001**	**0.008**
**Nephrolithiasis recurrence**			
LC9 (continuous)	0.970 (0.962,0.977) **<0.0001**	0.969 (0.960,0.978) **<0.0001**	0.978 (0.956,1.001) 0.06
LC9 (in quartiles)			
Quartile 1	Ref	Ref	Ref
Quartile 2	0.850 (0.571,1.265) 0.416	0.792 (0.533,1.178) 0.244	0.858 (0.573,1.286) 0.448
Quartile 3	0.620 (0.423,0.908) **0.015**	0.602 (0.406,0.892) **0.012**	0.682 (0.360,1.295) 0.234
Quartile 4	0.250 (0.165,0.377) **<0.0001**	0.257 (0.168,0.394) **<0.0001**	0.305 (0.159,0.586) **<0.001**
*P* for trend	**<0.0001**	**<0.0001**	**0.006**

OR, odds ratio; CI: confidence interval; LC9, Life’s Crucial 9; PIR, poverty income ratio; BMI, body mass index; CVD, cardiovascular disease.

^aNon-adjusted model: adjusted for none.^

^bMinimally adjusted model: adjusted for gender, age, race.^

^cFully adjusted model: adjusted for gender, age, race, PIR, BMI, education, marital status, smoking, alcohol, physical activity, gout, diabetes, hypertension, stroke, CVD and cancer.^

Statistically significant values were reported in bold.

**Figure 2:**
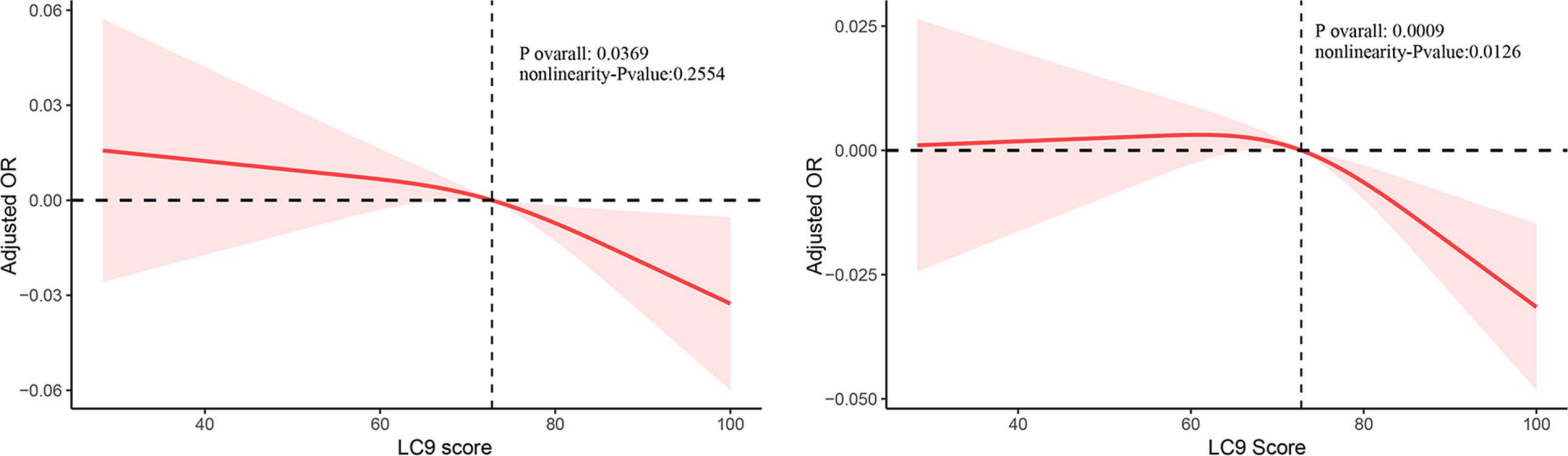
Restricted cubic spline of the association between LC9 score and kidney stone adjusted for all covariates.

The RCS analysis showed a negative linear relationship between LC9 and kidney stone occurrence (Figure 2A). In contrast, a nonlinear association was identified for kidney stone recurrence (Figure 2B). A comparison of standard and piecewise linear models revealed a significant LC9 effect (*p* < 0.05) in the likelihood ratio test. Using a piecewise linear model and recursive algorithm, the LC9 inflection point was determined to be 70.556 (Table 3). Beyond this threshold, each unit increase in LC9 corresponded to a 5.6% reduction in kidney stone risk (OR: 0.994, 95% CI: 0.914–0.972).

**Table 3:** Results of binary logistic regression and piecewise linear regression model^a^.

Outcome: Nephrolithiasis recurrence	Adjusted OR (95% CI)	*p*-value
Fitting by binary logistic regression model	0.98 (0.967–0.993)	0.003
Fitting by piecewise linear regression model		
Inflection point	72.777	
<70.556	0.994 (0.978–1.011)	0.481
>70.556	0.944 (0.914–0.972)	<0.001
*P* for likelihood ratio test		0.005

OR, odds ratio; CI: confidence interval; PIR, poverty income ratio; BMI, body mass index; CVD, cardiovascular disease.

^aAll models were adjusted for gender, age, race, PIR, BMI, education, marital status, smoking, alcohol, physical activity, gout, diabetes, hypertension, CVD and cancer.^

### 3.3 Analyses of subgroups and interactions

Subgroup analyses revealed that race modified the relationship between LC9 and kidney stones after adjusting for other covariates. The odds ratios for the Q4 group compared to Q1 were 0.281 for Mexican Americans, 0.223 for other Hispanics, 0.756 for non-Hispanic whites, 0.558 for non-Hispanic Blacks, and 0.223 for individuals of other races, with a *p*-value for interaction of 0.044. Additionally, both race and gout were identified as modifiers for kidney stone recurrence, with interaction *p*-values of 0.003 and 0.012, respectively (Supplementary Table S2).

## 4 Discussion

The LC9 score builds upon the LE8 framework, aiming to improve the development and evaluation of integrated cardiovascular care models that incorporate mental health management. This study investigates the association between the newly developed LC9 score and both the incidence and recurrence of kidney stones in adults. The results demonstrate that higher LC9 scores are inversely related to the risk of kidney stone formation. These findings highlight the potential benefit of further exploring integrated health behaviors and factors that may help prevent kidney stones.

Our study evaluated the predictive value of multidimensional LC9 measures in reducing the risk of kidney stones and highlighted the synergistic impact of depression on cardiovascular health. Previous research has shown that adequate fluid intake (13), a balanced diet (14), low fat (15), moderate physical activity (16), smoking cessation (17), maintaining normal sleep duration (18), and mental well-being are effective strategies to lower the risk of kidney stones (19). Our findings further underscore the importance of comprehensively assessing these behaviors.

Dietary factors significantly influence both the formation and recurrence of kidney stones. Research has shown that high consumption of calcium, oxalate, and animal protein increases the risk of kidney stone development (20). On the other hand, increasing fluid intake appropriately can dilute calcium oxalate, uric acid, and other urinary components, reducing the likelihood of stone formation (21). Physical activity may lower the risk of kidney stones by enhancing urinary metabolism. This effect is likely due to improved calcium metabolism and better bone health, which decrease calcium salt deposition in the urine (22). Depression, recently added as a component of the LC9 score, also merits further exploration in this context. It may indirectly contribute to kidney stone risk through lifestyle factors (e.g., poor diet, physical inactivity) and physiological changes, such as elevated stress hormone levels (19). BMI, blood glucose, non-HDL cholesterol, and blood pressure are also linked to kidney stone formation. Elevated BMI is associated with higher levels of uric acid and oxalate, increasing the risk of stone development. This study found that maintaining a healthy BMI significantly lowers the risk of kidney stones. Additionally, managing blood glucose and blood pressure may reduce the likelihood of stone formation by minimizing renal metabolic stress (23).

Stratified analysis identified an interaction between ethnicity and the LC9 score in relation to both kidney stone occurrence and recurrence. This interaction may result from various factors, including genetic predisposition, physiological differences, mental health, socioeconomic conditions, and cultural practices. Furthermore, an interaction was observed between gout and the LC9 score concerning kidney stone recurrence. Tailored interventions for gout patients, focusing on dietary management and mental health, could help lower the risk of recurrent kidney stones.

Our study underscores the potential of the LC9 score in fostering interdisciplinary collaboration, particularly between urologists and cardiologists (8, 24). By highlighting lifestyle factors associated with cardiovascular disease risk, the LC9 score serves not only as a predictor for cardiovascular conditions but also as a preventive tool for kidney stones. Integrating this score into routine health assessments could improve early detection and intervention. However, the cross-sectional nature of our research limits the ability to draw causal conclusions. Future studies should focus on larger prospective cohorts to validate the relationship between the LC9 score and kidney stone risk and to explore its clinical utility in personalized health management across diverse populations.

## 5 Conclusion

While our study demonstrates that higher LC9 scores are associated with a lower risk of kidney stones, further research is needed to clarify the causal relationship between LC9 and kidney stone formation. Prospective cohort studies and randomized controlled trials would provide stronger evidence to support these findings. Additionally, future research could explore how integrating the LC9 score into clinical practice might guide personalized prevention strategies for kidney stones, particularly in individuals at higher cardiovascular risk. These findings could also inform public health recommendations aimed at promoting cardiovascular health and preventing kidney stones through lifestyle modifications.

## Data Availability

The original contributions presented in the study are included in the article/Supplementary material, further inquiries can be directed to the corresponding authors.
