# Supplementary material for: Association between life’s crucial 9 and kidney stones: a population-based study
Source: Front Med (Lausanne). 2025 Mar 6;12:1558628. doi: 10.3389/fmed.2025.1558628 (PMC11922861; doi:10.3389/fmed.2025.1558628)
Supplement: Supplementary file 1 [file Table_1.docx]

**Table S1**. Definition and scoring approach for quantifying Life's Crucial 9

| **Domain** | **CVH Metric** | **Method of Measurement** | **Quantification of CVH Metric(≥20 Years)** |
| --- | --- | --- | --- |
| **Health Behaviors** | **Diet** | **Measurement**: Self-reported daily intake of a DASH-style eating pattern  **Example tools for measurement**: DASH diet score (populations) | Quantiles of DASH-style diet adherence  **Scoring (Population)**:  Points Quantile  100 ≥95^th^ %ile (top/ideal diet)  80 75^th^ – 94^th^ %ile  50 50^th^ – 74^th^ %ile  25 25^th^ – 49^th^ %ile  0 1^st^ – 24^th^ %ile (bottom/least ideal quartile) |
|  | **Physical activity** | **Measurement**: Self-reported minutes of moderate or vigorous physical activity per week  **Example tools for measurement**:  NHANES PAQ-K questionnaire | **Metric**: Minutes of moderate (or greater) intensity activity per week  **Scoring**:  Points Minutes  100 ≥150  90 120 – 149  80 90 – 119  60 60 – 89  40 30 – 59  20 1 – 29  0 0 |
|  | **Nicotine exposure** | **Measurement**: Self-reported use of cigarettes or inhaled nicotinedelivery system  **Example tools for measurement**:  NHANES SMQ | **Metric**: Combustible tobacco use and/or inhaled NDS use; or secondhand smoke exposure  **Scoring**:  Points Status  100 Never smoker  75 Former smoker, quit ≥5 yrs  50 Former smoker, quit 1 - <5 yrs  25 Former smoker, quit <1 year, or currently using inhaled NDS  0 Current smoker  Subtract 20 points (unless score is 0) for living with active indoor smoker in home |
|  | **Sleep health** | **Measuremen**t: Self-reported average hours of sleep per night  **Example tools for measurement**:  “On average, how many hours of sleep do you get per night?” Consider objective  sleep/actigraphy data from wearable technology, if available | **Metric**: Average hours of sleep per night  **Scoring**:  Points Level  100 7 – <9  90 9 - <10  70 6 - <7  40 5 - <6 or ≥10  20 4 - <5  0 <4 |
| **Health Factors** | **Body mass index** | **Measurement**: Body weight (kg) divided by height squared (m^2^)  **Example tools for measurement**:  Objective measurement of height and weight | **Metric**: Body mass index (kg/m^2^)  **Scoring**:  Points Level  100 <25  70 25.0 – 29.9  30 30.0 – 34.9  15 35.0 – 39.9  0 ≥40.0 |
|  | **Blood lipids** | **Measurement**: Plasma total and HDL-cholesterol with calculation of non-HDL-cholesterol  **Example tools for measurement**:  Fasting or non-fasting blood sample | **Metric**: Non-HDL-cholesterol (mg/dL)  **Scoring**:  Points Level  100 <130  60 130 – 159  40 160 – 189  20 190 – 219  0 ≥220  If drug-treated level, subtract 20 points |
|  | **Blood glucose** | **Measurement**: Fasting blood glucose or casual hemoglobin A1c  **Example tools for measurement**:  Fasting (FBG, HbA1c) or nonfasting (HbA1c) blood sample | **Metric**: Fasting blood glucose (mg/dL) or  Hemoglobin A1c (%)  **Scoring**:  Points Level  100 No history of diabetes and FBG <100 (or HbA1c < 5.7)  60 No diabetes and FBG 100 – 125 (or HbA1c 5.7-6.4) (Pre-diabetes)  40 Diabetes with HbA1c <7.0  30 Diabetes with HbA1c 7.0 – 7.9  20 Diabetes with HbA1c 8.0 – 8.9  10 Diabetes with Hb A1c 9.0 – 9.9  0 Diabetes with HbA1c ≥10.0 |
|  | **Blood pressure** | **Measurement**: Appropriately measured systolic and diastolic blood pressure  **Example tools for measurement**:  Appropriately sized blood pressure cuff | **Metric**: Systolic and diastolic blood pressure (mmHg)  **Scoring**:  Points Level  100 <120/<80 (Optimal)  75 120-129/<80 (Elevated)  50 130-139 or 80-89 (Stage I HTN)  25 140-159 or 90-99  0 ≥160 or ≥100  Subtract 20 points if treated level |
| **Psychological health** | **Depression** | **Measurement**: Patient Health Questionnaire‐9 (PHQ‐9) score  **Example tools for measurement**:  NHANES dpq questionnaire | **Metric**: (PHQ‐9) score  **Scoring**:  Points Level  100 0-4  75 5-9  50 10-14  25 15-19  0 20-27 |

**Table S2.** The Results of subgroup analysis^a^

| Subgroup | Nephrolithiasis |  |  | Nephrolithiasis recurrence |  |
| --- | --- | --- | --- | --- | --- |
|  | OR(95%CI), P-value | P for interaction |  | OR(95%CI), P-value | P for interaction |
| **Age category, n(%)** |  | 0.304 |  |  | 0.435 |
| 20-39 | 0.365(0.201,0.661) 0.001 |  |  | 0.200(0.052, 0.770) 0.021 |  |
| 40-59 | 0.959(0.542,1.696) 0.883 |  |  | 0.244(0.082,0.721) 0.012 |  |
| ≥60 | 0.506(0.284,0.904) 0.023 |  |  | 0.502(0.199,1.270) 0.141 |  |
| **Gender, n(%)** |  | 0.767 |  |  | 0.109 |
| Female | 0.752(0.466,1.213) 0.234 |  |  | 0.416(0.158,1.092) 0.073 |  |
| Male | 0.511(0.332,0.787) 0.003 |  |  | 0.222(0.101,0.489) **<0.001** |  |
| **Race, n(%)** |  | 0.044 |  |  | 0.003 |
| Mexican American | 0.281(0.100,0.791) 0.018 |  |  | 0.048(0.004,0.586) **0.019** |  |
| Non-Hispanic Black | 0.558(0.208,1.499) 0.238 |  |  | 1.590(0.364,6.944) 0.527 |  |
| Non-Hispanic White | 0.756(0.505,1.131) 0.168 |  |  | 0.330(0.161,0.676) 0.003 |  |
| Other Hispanic | 0.223(0.081,0.613) 0.005 |  |  | 0.022(0.002,0.297) 0.005 |  |
| Other Race | 0.261(0.066,1.041) 0.057 |  |  | 0.177(0.012,2.618) 0.200 |  |
| **PIR category, n(%)** |  | 0.862 |  |  | 0.997 |
| ≤1.3 | 0.544(0.284,1.039) 0.064 |  |  | 0.228(0.059,0.887) 0.034 |  |
| 1.3-3.5 | 0.566(0.297,1.082) 0.083 |  |  | 0.397(0.116, 1.362) 0.137 |  |
| ＞3.5 | 0.696(0.397,1.222) 0.200 |  |  | 0.264(0.114,0.615) 0.003 |  |
| **BMI category, n(%)** |  | 0.393 |  |  | 0.218 |
| <25 kg/m2 | 0.894(0.417,1.918) 0.767 |  |  | 0.360(0.118,1.101) 0.072 |  |
| 25-30 kg/m2 | 0.558(0.327,0.949) 0.032 |  |  | 0.172(0.065,0.460) <0.001 |  |
| ≥30 kg/m2 | 0.777(0.409,1.474) 0.429 |  |  | 0.646(0.183,2.275) 0.486 |  |
| **Marital status, n (%)** |  | 0.488 |  |  | 0.638 |
| Married/Living with partner | 0.561(0.385,0.816) 0.003 |  |  | 0.258(0.120,0.552) <0.001 |  |
| Divorced/ Separated/ Widowed | 0.805(0.380,1.709) 0.563 |  |  | 0.356(0.095,1.334) 0.121 |  |
| Never married | 1.034(0.251,4.255) 0.962 |  |  | 1.255(0.090,17.516) 0.862 |  |
| **Education, n(%)** |  | 0.247 |  |  | 0.056 |
| Less than 9th grade | 0.590(0.156, 2.234) 0.426 |  |  | 0.283(0.013, 5.977) 0.406 |  |
| 9–11th grade | 0.168(0.066,0.428) <0.001 |  |  | 0.060(0.005, 0.673) 0.024 |  |
| High school graduate | 0.613(0.295,1.273) 0.183 |  |  | 0.155(0.042,0.580) 0.007 |  |
| Some college | 0.775(0.461,1.302) 0.327 |  |  | 0.559(0.128,2.448) 0.430 |  |
| College or above | 0.706(0.351,1.420) 0.319 |  |  | 0.332(0.122, 0.906) 0.032 |  |
| **Smoke, n (%)** |  | 0.419 |  |  | 0.653 |
| Former | 0.509(0.274,0.947) 0.034 |  |  | 0.477(0.202, 1.125) 0.089 |  |
| Never | 0.853(0.578,1.260) 0.414 |  |  | 0.368(0.168,0.807) 0.014 |  |
| Now | 0.715(0.179,2.849) 0.625 |  |  | 0.578(0.081, 4.138) 0.576 |  |
| **Alcohol, n(%)** |  | 0.952 |  |  | 0.15 |
| Former | 0.503(0.222,1.142) 0.098 |  |  | 0.254(0.051, 1.262) 0.092 |  |
| Never | 0.431(0.196,0.948) 0.037 |  |  | 0.240(0.055, 1.045) 0.057 |  |
| Now | 0.666(0.446,0.996) 0.048 |  |  | 0.294(0.148,0.582) <0.001 |  |
| **Diabetes, n(%)** |  | 0.823 |  |  | 0.488 |
| No | 0.685(0.480,0.978) 0.038 |  |  | 0.326(0.161,0.660) 0.003 |  |
| Yes | 0.429(0.158,1.164) 0.094 |  |  | 0.215(0.045,1.037) 0.055 |  |
| **Hypertension, n(%)** |  | 0.428 |  |  | 0.44 |
| No | 0.660(0.395,1.104) 0.110 |  |  | 0.317(0.117, 0.859) 0.025 |  |
| Yes | 0.653(0.361,1.183) 0.154 |  |  | 0.310(0.123,0.785) 0.015 |  |
| **CVD, n(%)** |  | 0.823 |  |  | 0.701 |
| No | 0.685(0.480,0.978) 0.038 |  |  | 0.399(0.193,0.827) 0.015 |  |
| Yes | 0.429(0.158,1.164) 0.094 |  |  | 0.038(0.005, 0.286) 0.002 |  |
| **Gout, n(%)** |  | 0.326 |  |  | 0.012 |
| No | 0.696(0.494,0.980) 0.038 |  |  | 0.361(0.184,0.711) 0.004 |  |
| Yes | 0.255(0.046,1.411) 0.113 |  |  | 0.235(0.077,0.713) 0.012 |  |
| **Cancer, n(%)** |  | 0.59 |  |  | 0.577 |
| No | 0.634(0.438,0.916) 0.017 |  |  | 0.368(0.179,0.755) 0.008 |  |
| Yes | 0.578(0.200,1.673) 0.302 |  |  | 0.095(0.019, 0.485) 0.006 |  |

BMI body mass index, RIP ratio of family income to poverty, CVD cardiovascular disease;

^a^Adjusted for all potential confounding factors including age, race, education, sex, and RIP (ratio of family income to poverty), BMI (body mass index), smoking, hypertension, diabetes, gout, CVD, cancer. The model was not adjusted for the factor itself in each stratification
